# Enquiry Answered

**Published:** 1858-11

**Authors:** 


### ENQUIRY ANSWERED.

Dr. Davis, of the *Chicago Medical Journal*, asks, "who are the senior editors of the *Nashville* and *Peninsular Journals*, and who constituted them defenders of the American Medical Association?"

We can only answer for ourself. Our name is W. K. Bowling. We are relieved of the necessity of asking who is the senior editor of the *Chicago Medical Journal*, for that gentleman has published to the world that he is a member of the "Methodist church, north," and the papers of Chicago have, if we remember rightly, published that he is a defeated candidate for the mayoralty of the City of Chicago."

Dr. Davis claims to be the father of the American Medical Association, and we claim the right to defend the offspring even of such a father as he.

We hope Dr. Davis will regard his very civil question as most civilly and frankly answered.—*Nashville Journal.*

Not quite, Dr. Bowling. An answer that contains *three errors* to *one truth*, can scarcely be considered either "civil" or "frank." That the name of the senior editor of the *Nashville Journal* is W. K. Bowling no one will doubt who observes the amount of GAS which escapes every month through the editorial columns of that periodical. That I have ever "*published* to the world that I am a member of the 'Methodist church, NORTH,'" is wholly untrue; for my creed admits of no sectionalism, either in religion, politics or science. That "the papers of Chicago have published that I am a defeated candidate for the mayoralty of the city of Chicago," is equally untrue; for I have never been a candidate for the office of *Mayor* in this or any other city. That "Dr. Davis *claims* to be the father of the American Medical Association," is the *third error*. I have never made any such claim, either at home or abroad; in print or out of print. If others have applied that honorable title to me, it is their business, not mine. Will Dr. Bowling copy, and try again?

